# Salinity as an Abiotic Stressor for Eliciting Bioactive Compounds in Marine Microalgae

**DOI:** 10.3390/toxins16100425

**Published:** 2024-10-01

**Authors:** Adrián Macías-de la Rosa, Lorenzo López-Rosales, Antonio Contreras-Gómez, Asterio Sánchez-Mirón, Francisco García-Camacho, María del Carmen Cerón-García

**Affiliations:** 1 Department of Chemical Engineering, University of Almeria, 04120 Almeria, Spain; amd202@ual.es (A.M.-d.l.R.); acontre@ual.es (A.C.-G.); asmiron@ual.es (A.S.-M.); fgarcia@ual.es (F.G.-C.); mcceron@ual.es (M.d.C.C.-G.); 2 Research Centre on Mediterranean Intensive Agrosystems and Agri-Food Biotechnology (CIAIMBITAL), University of Almeria, 04120 Almeria, Spain

**Keywords:** microalgae, *Chrysochromulina rotalis*, *Amphidinium carterae*, *Heterosigma akashiwo*, valuable compounds, osmotic shock, salinity and two-stage cultivation

## Abstract

This study investigated the impact of culture medium salinity (5–50 PSU) on the growth and maximum photochemical yield of photosystem II (*Fv*/*Fm*) and the composition of carotenoids, fatty acids, and bioactive substances in three marine microalgae (*Chrysochromulina rotalis*, *Amphidinium carterae*, and *Heterosigma akashiwo*). The microalgae were photoautotrophically cultured in discontinuous mode in a single stage (S1) and a two-stage culture with salt shock (S2). A growth model was developed to link biomass productivity with salinity for each species. *C. rotalis* achieved a maximum biomass productivity (*Pmax*) of 15.85 ± 0.32 mg·L^−1^·day^−1^ in S1 and 16.12 ± 0.13 mg·L^−1^·day^−1^ in S2. The salt shock in S2 notably enhanced carotenoid production, particularly in *C. rotalis* and *H. akashiwo*, where fucoxanthin was the main carotenoid, while peridinin dominated in *A. carterae*. *H. akashiwo* also exhibited increased fatty acid productivity in S2. Salinity changes affected the proportions of saturated, monounsaturated, and polyunsaturated fatty acids in all three species. Additionally, hyposaline conditions boosted the production of haemolytic substances in *A. carterae* and *C. rotalis.*

## 1. Introduction

Photosynthetic microalgae are microorganisms that require light, carbon dioxide, and nutrients to grow properly. They are found in most aquatic ecosystems, and thanks to their metabolic plasticity, they can thrive under a wide variety of environmental conditions. This great capacity for adaptation, together with the ability to produce a wide range of substances with high added value, means they are microorganisms that can be utilised in bioengineering processes [1]. However, both their ability to adapt to different environments and the quantity and diversity of the bioproducts they generate are dependent not only on the microalgae species but also on the strain of microalgae used [2]. Therefore, it is crucial to carry out bioprospecting studies in which the response of different microalgae under different culture conditions is evaluated in order to select those species that offer the best yields in terms of producing metabolites of interest at the lowest costs. The most relevant abiotic factors involved in the cultivation of photoautotrophic microalgae are temperature, pH, irradiance, and the salinity of the culture medium [3].

Microalgae can perform various important functions in the environment as well as in daily human life. Accordingly, these microorganisms have been used in the cosmetic [4], pharmacological [5], biofuels [6], and food [7] sectors. Microalgae have also been used to treat urban wastewater [8] and effluents from industrial processes [9] and fish farms [10]. The use of microalgae in multiple sectors is a result of their great capacity to produce biomass over a short period of time and to generate very interesting biomolecules such as carotenoids, proteins, fatty acids, or poliketics with pharmacological applications [4,5]. However, due to the great biodiversity in the world of microalgae (there are more than 20,000 species [11]), individualized conditions need to be established regarding the culture, pH, temperature, lighting, and salinity (etc.) so as to maximize their growth and/or generate specific bioproducts [12].

The culture medium’s salinity is one of the most determining factors affecting the cellular growth, metabolism and productivity of marine microalgae biomolecules [13]. Salinity can affect microalgae in a variety of ways: (a) causing osmotic stress, (b) causing ion absorption or loss resulting in ionic stress, (c) causing changes in cellular ionic ratios, and (d) changing the size of cells through water absorption [14]. Establishing an optimal salinity value for a microalgae culture depends on the microalgae species; this is of great importance both in terms of growth and the production of biomolecules [15]. Exceeding this optimal salinity value can trigger hypersaline stress, whilst lower levels can cause hyposaline stress. In general, salt stress causes ionic imbalances, osmotic stress, and oxidative damage due to the production of reactive oxygen species (ROS). To mitigate the effects of salt stress, microalgae develop complex mechanisms ranging from the exclusion of harmful ions via transport systems [16], the synthesis of osmoregulatory substances such as glycerol [17] or mannitol [18], and the synthesis of antioxidant compounds to eliminate ROS [19].

Salt stress has been used in microalgae biotechnology to overproduce carotenoids [15], fatty acids [20], and other types of biomolecules of interest [21]. However, setting less than optimal salinity conditions usually reduces the growth of the microalgae, the biomass productivity, and, by extension, the bioproduct productivity. An effective strategy for obtaining high product yields and overproducing high-value-added biomolecules is to induce salt stress by applying a two-stage culture [22]. In Stage I, the microalgae are cultivated under optimal conditions, while in Stage II, a stress phase is implemented to increase metabolite production. This strategy has proven to be an effective and inexpensive way to overproduce carotenoids in microalgae [15]. For example, a two-stage culture was used to cultivate the marine microalga *Dunaliella salina* to produce β-carotene, achieving productivity of 0.35 mg·mL^−1^·day^−1^ after increasing the medium’s salinity in the second stage of the culture [23]. Regarding lipid production, numerous publications have demonstrated that the application of a two-stage culture, in which the salinity is varied in the second stage, has the effect of increasing or decreasing the microalgal lipid content [24,25,26].

The aim of this study is to investigate the effects of salinity variation on the growth and production of valuable compounds such as carotenoids, fatty acids, and bioactive molecules with haemolytic properties in three marine microalgae species: *Chrysochromulina rotalis*, *Amphidinium carterae,* and *Heterosigma akashiwo*. The growth data collected enabled the development of a mathematical model to predict the growth of each species based on the salinity of the culture medium. The research offers important insights into the behaviour of these microalgae in response to salinity fluctuations, similar to those found in photobioreactors, supporting their large-scale cultivation. Furthermore, this study assesses the effectiveness of a two-phase cultivation technique in enhancing the production of the previously described valuable compounds compared to batch cultivation.

## 2. Results and Discussion

### 2.1. Influence of Salinity on Culture Growth

**Figure 1:**
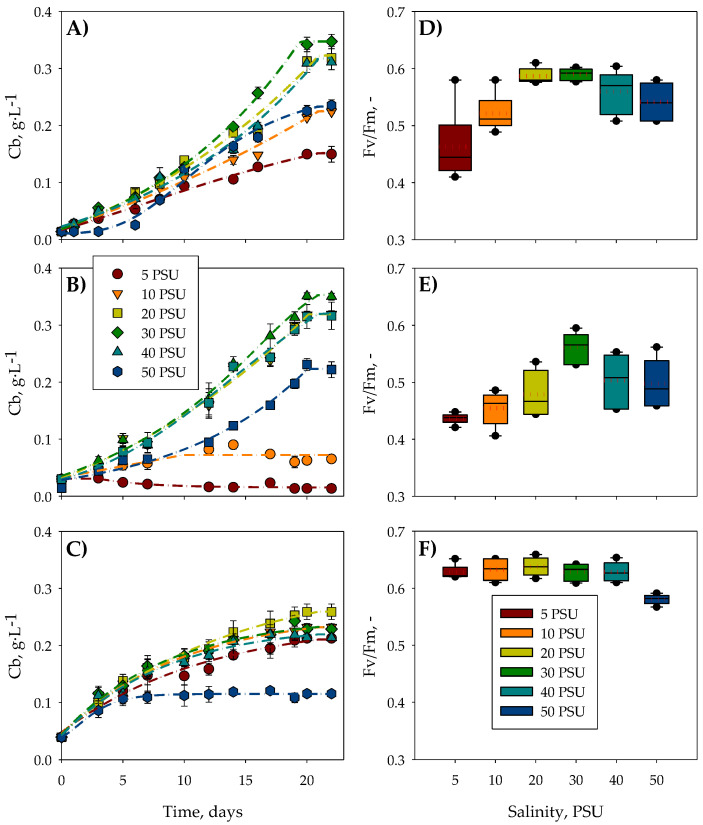
Biomass growth curves for the microalgae (**A**) *C. rotalis*, (**B**) *A. carterae*, (**C**), and *H. akashiwo* and the *Fv*/*Fm* for (**D**) *C. rotalis*, (**E**) *A. carterae* and (**F**) *H. akashiwo* in S1. The red dotted line in the *Fv*/*Fm* graph represents the average value.

Figure 1 shows the temporal evolution of the biomass (*Cb*) as a function of the most representative salinities tested (5, 10, 20, 30, 40, and 50 PSU) during each microalga’s Stage I (S1) culture. The growth kinetics described by the three species follow a typical microbial pattern, characterized by an initial adaptation phase to the new culture conditions, followed by an exponential growth phase, and concluding with a stationary phase. *C. rotalis* and *A. carterae* (Figure 1A,B) presented adaptation phases of 1 or 2 days at almost all the salinities, except for 30 PSU, in which growth was immediate because this was the salinity from which the inoculum originated. After 20 days of culture, these microalgae species reached the stationary growth phase in all the tests carried out as a result of consuming practically all the nitrates and phosphates in the culture medium (Table 1). Conversely, *H. akashiwo* did not present an adaptation phase at any of the salinities tested, reaching the stationary phase at around day 17 for salinities between 5–40 PSU. For values above 40 PSU, growth was significantly inhibited, with the microalga reaching the stationary growth phase on day 7 in the 50 PSU trial. 

Measuring the maximum photochemical yield of photosystem II (*Fv*/*Fm*) by using pulse amplitude modulation (PAM) fluorometry is a non-invasive technique that has been used as a thermal, saline, nutritional, and light stress indicator in phytoplankton [27]. As can be seen in Figure 1D–F, the *Fv*/*Fm* values of the three microalgae modulated in response to the salinity of the medium in which they were cultured. Both *C. rotalis* and *A. carterae* (Figure 1D,E) experienced an increase in the mean *Fv*/*Fm* value (the dotted red line), reaching a maximum value close to 0.6 at 20 PSU for *C. rotalis* and 30 PSU for *A. carterae*, followed by a decrease above those salinities. However, *H. akashiwo* (Figure 1F) maintained a constant mean *Fv*/*Fm* value of 0.65 without significant variations (*p* > 0.05) up to a salinity of 40 PSU, above which it decreased to 0.5. Although none of the three microalgae species reached *Fv*/*Fm* values considered photosynthetically unhealthy [28], notable differences were observed between the mean *Fv*/*Fm* values at optimal saline concentrations and those at the other salinities. It is important to note that, in general, greater variability in the *Fv*/*Fm* values was detected under non-optimal salinity conditions, which shows the gradual deterioration of the cells as the days passed following their inoculation in media with non-ideal saline concentrations. Masojídek et al. (2000) [29] observed a notable decrease in the *Fv*/*Fm* value in the microalgae *Chlorococcum* sp. when it was subjected to salt stress. In addition, they highlighted that this value progressively decreased as the days went by under these stressful conditions.

**Figure 2:**
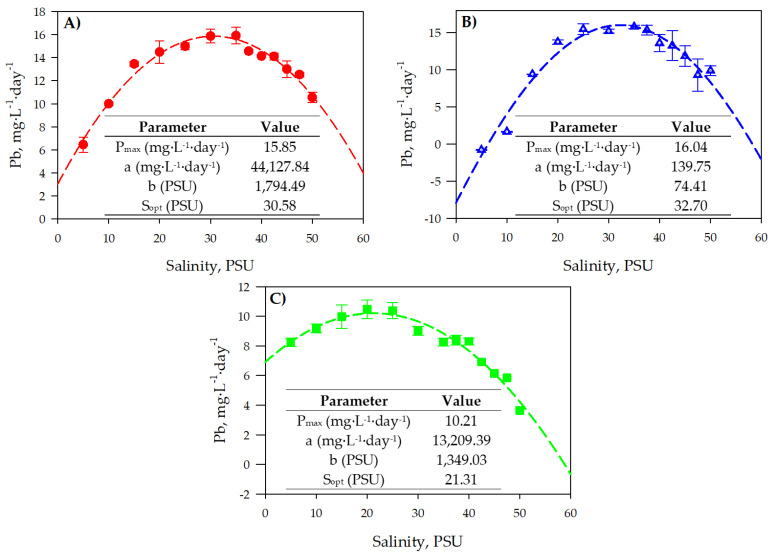
Biomass productivity curves for the microalgae in S1 (**A**) *C. rotalis*, (**B**) *A. carterae* and (**C**) *H. akashiwo* and the model fitting results achieved in the different experiments. Data points are averages, and lines are the model (Equation (3)) that adequately fits the different experimental sets performed. The parameters and their values that fit the data are shown within the figures.

To establish any microalgae-based bioprocess at the industrial scale, it is essential to have mathematical models that allow one to predict the growth of these microorganisms based on factors such as temperature, light, pH, nutrient concentration, and salinity. Although there are numerous growth models that consider variables such as temperature, irradiance, pH, etc. [30], to date, only polynomial correlations between growth and medium salinity have been developed [31], or models originally designed for other variables, such as temperature, which have been adapted to analyze the impact of salinity [32]. Figure 2 shows the final biomass productivity (*Pb*) as a function of the salinities tested in S1, as well as the adjustment parameter values of the proposed growth model for the three microalgae. As can be observed, *C. rotalis* (Figure 2A) experienced a *Pb* variation ranging from a minimum value of 6.16 ± 1.11 mg·L^−1^·day^−1^ at 5 PSU to a maximum of 15.19 ± 0.46 mg·L^−1^·day^−1^ at 35 PSU. The optimal salinity value, determined by adjusting the growth model, was 30.99 PSU, a value very similar to that existing on the coasts of the Cantabrian Sea, Norway, and the Mediterranean Sea—habitats where *C. rotalis* usually proliferates. This relationship between growth and medium salinity, including the optimal salinity value obtained for *C. rotalis*, was observed in the haptophyte *Isochrysis galbana* [33]. When cultivated at different salinities, it reached its maximum growth at 30 PSU. On the other hand, *A. carterae* (Figure 2B) suffered an inhibition in growth at low salinities, registering negative *Pb* values at salinities below 10 PSU. Salinity values above 10 PSU led to greater growth, reaching a maximum potential *Pb* value of 16.91 ± 0.28 mg·L^−1^·day^−1^ at a salinity of 32.27 PSU according to the proposed model. Morton et al. (1992) [34] observed that growth was maximal at a salinity of 34 PSU after cultivating *Amphidiniun klebsii* at different salinities, a value very close to that in our trials for *A. carterae*. However, a recent study in which only three salinity levels were evaluated found that *A. carterae* reached the highest *Cb* values at a salinity of 20 PSU [35]. *H. akashiwo*, on the other hand, presented good tolerance to low salinities (Figure 2C), reaching a *Pb* value of 10.12 ± 0.20 mg·L^−1·^day^−1^ at 5 PSU, a practically identical value (*p* > 0.05) to the maximum potentially achievable biomass productivity value (10.14 ± 0.47 mg·L^−1^·day^−1^ reached at 21.30 PSU), according to the model. From 25 PSU upwards, the *Pb* value decreased until it reached its lowest value at 50 PSU (5.50 ± 0.25 mg·L^−1^·day^−1^). The results obtained for *H. akashiwo* are in line with previous publications looking at this microalga. Haque et al. (2002) observed that growth was maximal at a salinity of 25 PSU after cultivating *H. akashiwo* at different salinities [36]. Conversely, Martínez et al. (2010) [37] set the optimal salinity range for *H. akashiwo* growth at 20–35 PSU after culturing six different strains. Table 1 presents the values of the mean square error (*RMSE*), the bias factor (*B_f_*), the accuracy factor (*A_f_*), and the correlation coefficient (*r*^2^) obtained by comparing the experimental data with the values proposed by the growth model for the three microalgae used in this study. To validate the proposed model’s use in cultivating other microalgae, it was also utilised to evaluate growth (as a function of salinity) for two further microalgae, *Alexandrium pacificum* [38] and *Desmodesmus* sp. [39], as well as for the halotolerant cyanobacterium *Arthrospira platensis* [40]. As can be observed in Table 2, the results indicate that the growth model based on the culture with medium salinity fits well with the experimental data. To use the proposed model for *A. platensis*, it was necessary to apply the decimal logarithm to the salinity values because, being a halotolerant cyanobacterium, it showed very small *Pb* responses to large changes in salinity. The *RMSE* values were less than 1 in all cases [41], indicating that the model accurately predicts the actual values. In addition, the *B_f_* and *A_f_* values were very close to 1, demonstrating a high degree of model adequacy [42].

**Figure 3:**
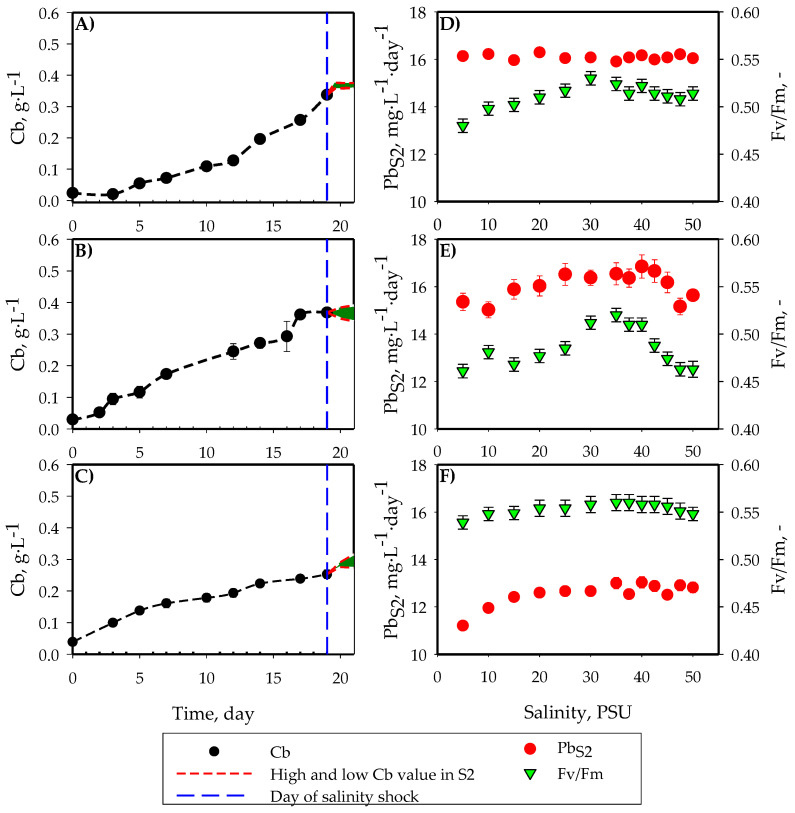
Culture growth kinetics, including the batch culture (S1) and the culture experiencing salt shock (S2) for (**A**) *C. rotalis*, (**B**) *A. carterae* and (**C**) *H. akashiwo*, along with the biomass productivity achieved in S2 for (**D**) *C. rotalis*, (**E**) *A. carterae* and (**F**) *H. akashiwo*. The blue line indicates the day on which the salt shock occurred; the red lines show the upper and lower limits of the *Cb* reached in this stage.

The *Cb* results, biomass productivity (*Pb_S_*_2_) and *Fv*/*Fm* obtained in S2, are shown in Figure 3. In these two-stage cultures, once the stationary growth phase was reached after 17 days of growth in discontinuous mode using the optimal salinity value obtained in S1, the salinity was suddenly changed, thus beginning the second stage of the culture, which lasted for 48 h. During the first stage of the culture, the three microalgae presented a growth pattern that was very similar to that obtained in S1 under optimal salinity conditions. Once the saline shock occurred, the *Cb* values in the second stage did not present significant differences between the maximum and minimum values (*p* < 0.05) in the case of *C. rotalis* (Figure 3A), thus resulting in a small variation in the *Pb_S_*_2_ values (Figure 3D). However, for *A. carterae* (Figure 3B), a variation was observed between the *Cb* maximum and minimum once the salinity changed. As can be seen in Figure 3E, significant variations (*p* > 0.05) were observed in the final *Pb_S_*_2_ values. The minimum *Pb_S_*_2_ values were recorded at the salinity extremes (<10 PSU and >45 PSU), with the best growth values being obtained at a salinity of 40 PSU. Finally, in Figure 3F, in the case of *H. akashiwo*, a direct relationship was established between the salinity of the shock and the *Pb_S_*_2_ up to 20 PSU, after which the increase in salinity did not produce a change in the *Pb_S_*_2_ value (*p* < 0.05).

### 2.2. Influence of Salinity on Carotenoid Production

**Figure 4:**
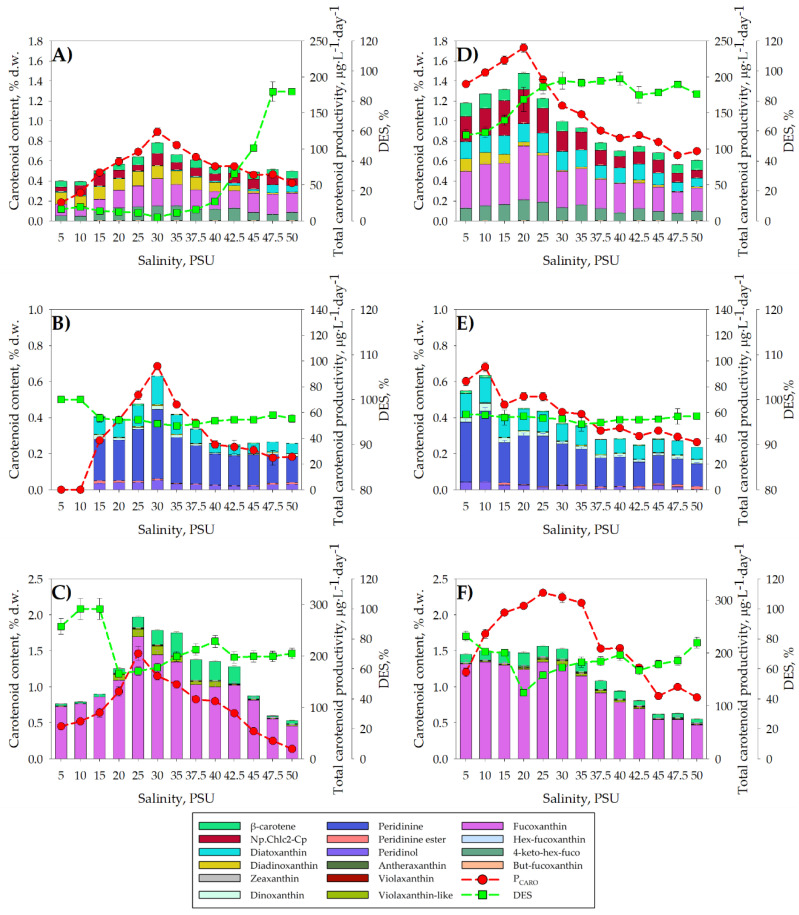
Influence of the salinity concentration (5–50 PSU) on the carotenoid profile expressed as a percentage of biomass dry weight (% d.w.) and the total carotenoid productivity expressed in μg·L^−1^·day^−1^ in Stage 1 (S1) for (**A**) *C. rotalis*, (**C**) *A. carterae* and (**E**) *H. akashiwo*. Variation in the carotenoid profile in Stage 2 (S2) in the biomasses harvested at the salinities indicated the three microalgae tested: (**B**) *C. rotalis*, (**D**) *A. carterae* and (**F**) *H. akashiwo*. The vertical bars indicate the average values of the cultures in duplicate and the analysis in triplicate, together with their standard deviation (SD). The red dots indicate the average total carotenoid productivity values, while the green dots indicate the average DES values with their error bars, both calculated as described in Section 4.

Different environmental parameters, such as temperature, irradiance, photoperiod duration, pH, nutrient concentration, salinity, etc., can affect the production of carotenoids in microalgae [43]. Figure 4 shows the carotenoid profiles and total carotenoid yields (*P_CARO_*) obtained for the three microalgae cultured at different salinities in S1 and S2. In Figure 4A,D, the carotenoid profile of *C. rotalis* is consistent with previous studies in which it was shown that this microalgae produced fucoxanthin and its derivatives, such as 4-keto-19′-hexanoyloxyfucoxanthin(4-keto-hex-fucoxanthin) and 19′-hex-fucoxanthin (hex-fucoxanthin), diadinoxanthin, diatoxanthin, nonpolar chlorophyll (similar to c2 of *C. rotalis*), and β-carotene [44]. The *Pc_ARO_* value of *C. rotalis* (Figure 4A,D) increased as salinity increased, reaching a value of 123.88 ± 5.74 μg·L^−1^·day^−1^ at 30 PSUs in S1, and up to 240.59 ± 5.74 μg·L^−1^·day^−1^ at 20 PSUs in S2. With the use of a two-stage cultivation strategy, it was possible to double the Pc_ARO_ value. Fucoxanthin is usually the main pigment produced by haptophytes [44], and this has aroused great interest in recent years due to its many bioactivities, such as its anti-diabetic, anti-obesity, antioxidant [45], and anti-cancer properties [46]. In the case of the haptophyte *C. rotalis*, it was observed that fucoxanthin and its derivatives made up the highest percentage of pigments of the total carotenoids present in the two strategies studied (S1 and S2), representing, on average, 50.75 ± 6.23% (S1) and 49.93 ± 4.32% (S2). These percentage values of fucoxanthin and its derivatives are comparable to the 40.3 ± 7.7% of the total carotenoids that fucoxanthin represented when *C. rotalis* was cultured in an artificially illuminated 80 L tubular photobioreactor (T-PBR) [47]. The maximum fucoxanthin productivity values achieved in the cultures were 39.75 ± 0.23 μg·L^−1^·day^−1^ (S1) and 78.76 ± 1.23 μg·L^−1^·day^−1^ (S2)—both maximums were reached at 20 PSU. Many microalgae have pigments such as diatoxanthin (Dtx) and diadinoxanthin (Ddx), which can interconvert with each other and, when integrated into the xanthophyll cycle, play a very important role in reducing oxidative processes [48]. The interconversion between these two pigments can be evaluated by calculating the de-epoxidation (DES) of Ddx to Dtx. The DES value can vary in response to different factors that cause oxidative stress, such as saline, light, or thermal stress, among others [29]. A high DES value indicates greater Dtx formation, which carries out antioxidant functions and is usually synthesized in response to the generation of ROS in the face of any type of abiotic stress [49]. As can be seen for *C. rotalis*, in the S1 cultures at salinities above 47.5 PSU, high DES values were reached, which could suggest high ROS generation. In the case of the S2 culture, a gradual increase in the DES value was observed, going from 57.21 ± 3.52% (at 5 PSU) up to values above 80% from 20 PSU. This increase in the DES value, with respect to the values recorded in S1, could be caused to a large extent by stress produced during the centrifugation and resuspension of cells carried out on day 17 of the S2 cultures.

In both strategies (Figure 4B,E), *A. carterae* synthesized diadinochrome, β-carotene, peridininol, diatoxanthin, dinoxanthin, diadinoxanthin, and peridinin, a carotenoid profile consistent with previous publications on this species [50]. In Figure 4B,E, the maximum *Pc_ARO_* values in the two strategies used were very similar, 96.08 ± 2.24 μg·L^−1^·day^−1^ for S1 at 30 PSU and 95.46 ± 3.18 μg·L^−1^·day^−1^ at a salinity of 10 PSU in S2. In contrast to *C. rotalis*, no improvement in the percentage of total carotenoids was observed in *A. carterae* when implementing a two-stage culture. The minimum and maximum values of the total carotenoid content ranged from 0.24 ± 0.01% d.w. (50 PSU) to 0.63 ± 0.01% d.w. (30 PSU) for S1 and between 0.23 ± 0.01% d.w. (50 PSU) and 0.64 ± 0.02% d.w. (10 PSU) for S2. The minimum values of total carotenoids produced in both strategies are comparable to the 0.359% d.w. and the 0.33% d.w. obtained, respectively, by Johansen et al. (1974) [51] and Molina Miras et al. (2018) [52] for *A. carterae*. As for the maximum carotenoid values generated by *A. carterae* in our study, they are far from the 1.34% d.w. reported for this same species when it was grown in an artificially illuminated 33L raceway photobioreactor (RW-PBR) [52]. However, our results are comparable to the 0.75% d.w. obtained by Kichouh et al. (2024) [53] when cultivating *A. carterae* in the discontinuous mode using T-flasks, a culture system similar to that used in our experiments. The main pigment synthesized by A. carterae, regardless of the salinity and culture strategy followed, was peridinin, reaching maximum values of 0.38 ± 0.01% d.w. (30 PSU) in S1 and 0.39 ± 0.01% d.w. (10 PSU) in S2. Peridinin has previously been reported as the main carotenoid present in *A. carterae* [51], constituting up to 0.2–0.9% d.w. [52], values similar to those achieved in our trials. Given the ability of *A. carterae* to produce peridinin, it has been proposed as a natural source of this pigment for the production of analytical standards [52]. Regarding DES, values above 90% were achieved in all the tests carried out, both in S1 and S2. The DES value can be affected by abiotic factors, the culture’s growth phase [54], and the concentration of nutrients [55]. Although the DES values achieved in our trials are unusually high, it is true that the cells harvested in S1 were in the stationary growth phase and nutritionally deficient, which added to the salt stress and might account for these high values. In Figure 3E, there was hardly any growth in S2, meaning there was a nutritional deficit—added to the stress caused by the S2 centrifugation, this could again explain the high DES values. Despite the DES values being high, it is true that in both culture strategies, the minimum DES value was reached at salinities close to the optimum for *A. carterae* determined in S1 (35 PSU).

As can be seen in Figure 4E,F, *H. akashiwo* produced fucoxanthin and its derivatives, such as hex-fucoxanthin, violaxanthin and its derivatives, antheraxanthin, zeaxanthin, and β-carotene pigments, all of which are typically present in this microalgae [37]. The maximum values of *Pc_ARO_* were 204.98 ± 1.24 μg·L^−1^·day^−1^ in S1 and 313.43 ± 2.24 μg·L^−1^·day^−1^ in S2; both of these values reached a salinity of 25 PSU. In S1 at 25 PSU, the total amount of carotenoids generated reached 1.97 ± 0.07% d.w., while in S2 at 25 PSU, the amount of total carotenoids was 1.56 ± 0.08% d.w. The values of the total carotenoid content are considerably higher than the 0.65 ± 0.05% d.w. obtained in a culture of *H. akashiwo* performed in a 10 L bubble-column photobioreactor (BC-PBR) [56]. The main difference between our experiments and the *H. akashiwo* cultivated in the BC-PBR system lies in the salinity of the culture medium. In our trials, the maximum carotenoid production values were reached at a salinity of 25 PSU, while in the BC-PBR system, the experiments were carried out at a salinity of 30 PSU. Fucoxanthin and violaxanthin have been reported as the predominant carotenoids in this species [37]. In all our trials, fucoxanthin was the main pigment, representing up to 91.7 ± 0.07% of the total carotenoid content in S1 at 5 PSU and 80.37 ± 0.07% in S2 at 10 PSU. The maximum productivity of fucoxanthin in S1 was 149.70 ± 2.24 μg·L^−1^·day^−1^ at 25 PSU, similar to the 160 μg·L^−1^·day^−1^ obtained when *H. akashiwo* was grown in a continuous mode in a BC-PBR [56]. However, the maximum productivity value of fucoxanthin in S2, 233.50 ± 0.23 μg·L^−1^·day^−1^, was clearly higher than that obtained in the culture carried out in the BC-PBR. Based on this productivity result, the implementation of a two-phase culture of *H. akashiwo*, in which salinity was varied, provided an apparent improvement in fucoxanthin productivity. Unlike *C. rotalis* and *A. carterae*, *H. akashiwo* contains zeaxanthin and violaxanthin as constitutive pigments integrated into the xanthophyll cycle [48]. In S1, DES values of 100% were reached at low salinities, while the lowest value was reached at 20 PSU (DES = 56.77± 1.54%), increasing its value again by increasing the salinity from 20 PSU, but without exceeding a value of 80% at any time. On the other hand, in S2, the highest DES values were obtained at 5 PSU (81.89 ± 0.4%) and 50 PSU (77.59 ± 0.06%), dropping to a minimum again at 20 PSU (44.07 ± 0.7%).

### 2.3. Influence of Salinity on Fatty Acids

The microalgal lipid content can be affected by the conditions in which the culture is grown, i.e., the concentration of nutrients in the medium, the light intensity, the temperature, the salt concentration, and the concentration of metals such as iron [43]. The lipid profile obtained from *C. rotalis* biomass showed the presence of saturated fatty acids (SFAs) such as 14:0, 16:0, and 18:0; monounsaturated fatty acids (MUFAs) such as 16:1n7, 18:1n7, and 20:1n9; and long-chain polyunsaturated fatty acids (PUFAs) such as 18:4n3, 20:5n3 (EPA), and 22:6n3 (DHA). This fatty acid profile is consistent with that previously reported for this species [47]. *A. carterae* produced the fatty acids 14:0, 16:0, 18:0, 18:1n9, 20:1n9, 18:4n3 (SDA), 20:5n3 (EPA), and 22:6n3 (DHA). This lipid profile is typically reported for *Amphidinium* [52]. *H. akashiwo* synthesized the fatty acids 14:0, 16:0, 16:1n7, 18:4n3 (SDA), 20:5n3 (EPA), and 22:6n3 (DHA), constituents previously reported for this microalgae [56]. 

**Figure 5:**
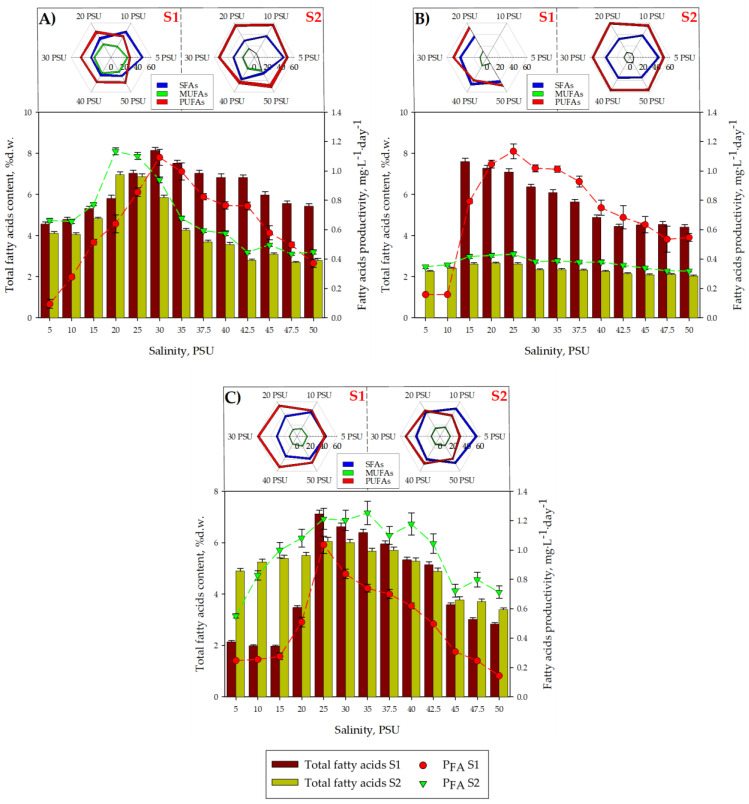
Radar plot of the saturated fatty acid (SFA), monounsaturated fatty acid (MUFA), and polyunsaturated fatty acid (PUFA) groups for each S1 and S2 stage and salinity in the three strains tested (**A**) *C. rotalis*, (**B**) *A. carterae* and (**C**) *H. akashiwo*. Influence of the salinity concentration (5–50 PSU) on the carotenoid profile expressed as a percentage of biomass dry weight (% d.w.) and the total fatty acid productivity expressed in mg·L^−1^·day^−1^ in Stage 1 (S1; brown bars) and in Stage 2 (S2; green vertical bars) for (**A**) *C. rotalis*, (**B**) *A. carterae* and (**C**) *H. akashiwo*. The vertical bars indicate the average values of the cultures in duplicate and the analysis in triplicate, together with their standard deviation (SD). The red dots indicate the average values of total fatty acid productivity (*P_FA_*) in S1, while the green dots indicate the average values of total fatty acids in S2 with their error bars.

As can be seen in Figure 5, the fatty acid content, both in S1 and S2, varied depending on the salinity, regardless of the microalgae studied. Modulation in the production of fatty acids and other biomolecules as a function of salinity was previously described for *Chlorella vulgaris*, *Tetraselmis chuii* and *Isochrysis galbana* [57]. It is striking, however, that in our study, no improvement in the total fatty acid content was observed in S2 compared to S1, with the exception of *H. akashiwo* at salinities < 20 PSU. This behaviour is similar to that observed in previous studies after applying hypersaline shock in cultures of certain marine microalgae, such as Dunaliella salina [25]. Not only did lipid production not increase, but it even dropped by 10% compared to optimal salinity conditions. In the case of *Isochrysis galbana*, it was observed that the application of hyposaline shock resulted in an increase in the percentage of total lipids, reaching 47% d.w. [58]. 

Regarding the total productivity of fatty acids (*P_FA_*) for *H. akashiwo*, in Figure 5C, the maximum *P_FA_* value reached in S2 (with respect to S1) increased from 0.74 ± 0.05 mg·L^−1^·day^−1^ (at 25 PSU) in S1 up to 1.25 ± 0.08 mg·L^−1^·day^−1^ (at 35 PSU) in S2. The increase in *P_FA_* at salinities < 20 PSU observed in *H. akashiwo* when carrying out a two-phase culture (S2) was motivated by an increase in the total fatty acid content. Meanwhile, at salinities > 20 PSU, the increase in the *P_FA_* value in S2 (compared to S1) was due to an increase in the biomass productivity experienced between S1 and S2. Regardless of the culture medium’s salinity or the strategy used, the maximum *P_FA_* values recorded for *H. akashiwo* in our study did not exceed the 6.5 mg·L^−1^·day^−1^ obtained when this microalgae was cultured in a 10 L BC-PBR [56]. In the case of *C. rotalis* (Figure 5A), the maximum *P_FA_* values reached in S1 and S2 were the same (*p* > 0.05), 1.29 ± 0.05 mg·L^−1^·day^−1^ (at 30 PSU) in S1 and 1.13 ± 0.08 mg·L^−1^·day^−1^ (at 20 PSU) in S2. These maximum *P_FA_* values are comparable to the 1.71 mg·L^−1^·day^−1^ obtained in previous studies when *Chrysochromulina* sp. was cultured in volumes of 250 mL in discontinuous mode [59]. The maximum *P_FA_* value in S2 was much lower than that reached in S1 in the case of *A. carterae* (Figure 5B), going from a value of 1.10 ± 0.06 mg·L^−1^·day^−1^ (at 25 PSU) in S1 to 0.43 ± 0.01 mg·L^−1^·day^−1^ (at 25 PSU) in S2. When comparing the maximum *P_FA_* value obtained for *A. carterae* in our trials, 1.10 ± 0.06 mg·L^−1^·day^−1^ (at 25 PSU) in S1, with the 2.19 ± 0.55 mg·L^−1^·day^−1^ reported for the same species cultured in a 33 L RW-PBR [52], a notable difference is observed. This discrepancy in the *P_FA_* values of A. carterae can be attributed to the differences in irradiance and culture medium between our assays and the culture performed in the RW-PBR since both the fatty acid profile and its productivity are strongly influenced by light [60] and nutrients [61]. However, a recent study in which *A. carterae* was cultured in batch mode using f/2 with a molar N/P ratio of 5 as the culture medium [53] obtained maximum *P_FA_* values of approximately 1.5 mg·L^−1^·day^−1^, close to those in our trials.

Regarding the percentage of SFAs present in the fatty acids contained in the *C. rotalis* biomass (Figure 5A), a decrease was observed with increasing salinity, going from 47.5 ± 1.7% (at 5 PSU) to 31.96 ± 2.7% (at 50 PSU) of the total fatty acids in S1, and from 44.3 ± 1.7% (at 5 PSU) to 27.47 ± 2.7% (at 50 PSU) of the total fatty acids in S2. Regarding the percentage of PUFAs present in the total fatty acids of *C. rotalis*, an increase from 28% (at 5 PSU) to 43% (at 50 PSU) was observed in S1. However, in S2, the average percentage of PUFAs remained relatively constant (*p* < 0.05) at 50% for the different salinities. The percentage of MUFAs, on the other hand, remained practically constant (*p* < 0.05) in S1 at an average value of 20%, while in S2, they increased from 5% (at 5 PSU) to 23% (at 50 PSU). Decreases in the SFA percentage accompanied by increases in MUFAs and/or PUFAs can be seen as a metabolic response by some microalgae to preserve cellular integrity against a saline change in the environment [62]. In the case of *C. rotalis*, regardless of the salinity and culture strategy, the main fatty acid in the SFAs was 14:0, while in the MUFAs, the predominant fatty acid was 18:1n9, although it did not represent more than 1% of the biomass in S1 (at 50 PSU).

It is important to note that the three microalgae used in this study, *C. rotalis* [47], *A. carterae* [52], and *H. akashiwo* [56], are capable of synthesizing PUFAs that have a marked antitumor capacity, such as 18:4n3, 20:5n3 (EPA) and 22:6n3 (DHA). In this sense, the DHA content present in the *C. rotalis* biomass increased with salinity in both S1 and S2, reaching 1.03 ± 0.05% d.w. (12.63% of the total fatty acids) in S1 at 30 PSU and 0.89 ± 0.05% d.w (12.85% of the total) for S2 at 20 PSU. On the other hand, the EPA concentration did not exceed 0.28 ± 0.01% d.w. (3.52% of the total) in S1 at 30 PSU or 0.08 ± 0.01% d.w. (1.01% of the total) in S2 at 20 PSU. The EPA percentage values, with respect to the total fatty acids obtained in our assays for the microalgae *C. rotalis*, are comparable to the 1.2% d.w. reported by González Cardoso et al. (2023) [47]. However, the maximum DHA percentage recorded by these authors was 5.7%, a value lower than the 12.85% achieved in S2 at 20 PSU. In the case of cultures made with *A. carterae*, the SFA percentage, with respect to the total fatty acids (Figure 5B) in S1, increased with increasing culture medium salinity, fixing the value of SFAs at 41% of the total fatty acids at 50 PSU. However, the SFA percentage in S2 decreased from 43% (at 5 PSU) to 34% at 20 PSU, not varying significantly (*p* < 0.05). The PUFA content in the total fatty acids of *A. carterae* decreased from 53% (at 15 PSU) to 48% (at 50 PSU) in S1, while in S2, the PUFA percentage did not vary significantly (*p* < 0.05), remaining at 55% over the range of salinities tested. The SFA content values were higher than the 18.8–20.4% reported for this species [63], although the PUFA percentage was similar to the 56% of total fatty acids obtained in cultures of *A. carterae* in a 33L RW-PBR [64]. The accumulation of SFAs as reserve substances (observed in S1) is a stress-adaptation strategy used by microalgae [64], which is detrimental to the production of unsaturated fatty acids. Regarding the distribution of the different *A. carterae* fatty acids in the SFA, MUFA, and PUFA fractions—for any salinity and regardless of the culture strategy used—the main fatty acid in the SFAs was 16:0, in the MUFAs, it was 18:1n9; and in the PUFAs, it was 22:6n3 (DHA) in S1. In S2, 20:5n3 predominated at low salinities and DHA at high salinities in the PUFA fraction. The percentage of EPA and DHA in the fatty acids of *A. carterae* reached 17.35 ± 0.20% (at 35 PSU) and 26.74 ± 0.20% (at 15 PSU) of the total fatty acids in the culture during S1 and up to 17.70 ± 0.07% (at 25 PSU) and 17.43 ± 0.20% (at 50 PSU) of the total during S2. These patterns for the main lipid profiles and fatty acids produced in SFAs, MUFAs, and PUFAs accord with what has previously been reported in *A. carterae* cultivation [52]. Finally, in Figure 5C, for *H. akashiwo*, it was observed that the SFA, MUFA, and PUFA profiles (referring to the total fatty acids in both S1 and S2) showed variations in the percentage of SFAs between 30 and 55%, variations in MUFAs between 11 and 17%, and variations in PUFAs between 29 and 58%. These percentages, referring to the total SFAs, MUFAs, and PUFAs, are comparable to the 30.7%, 22.2%, and 40.4%, respectively, that were previously reported for the species [56]. Based on the tests carried out on the microalgae *H. akashiwo*, the main SFA was found to be 16:0, the main MUFA was 18:1n9, and the main PUFA was 18:4n3 at low salinities in S1, while EPA was the predominant fatty acid at salinities greater than 20 PSU in S1 and at any salinity in S2. The EPA accounted for up to 25.53 ± 0.20% (at 30 PSU) and 23.72 ± 0.20% (at 35 PSU) of the total fatty acids in S1 and S2, respectively. These lipid profiles and fatty acid percentages coincide with previous studies [21] in which it was observed that for this same microalgae species, the fatty acid 16:0 was predominant at 20.95% of the SFAs, while EPA represented 13.04% of the PUFAs.

### 2.4. Influence of Salinity on the Haemolytic Activity of Microalgae

In addition to producing a wide variety of interesting carotenoids and fatty acids, these three microalgae have been reported as being producers of bioactives [4], making them attractive for setting up a possible microalgae-based bioprocess for pharmaceutical, cosmetic, or food supplement purposes. Recently, González Cardoso et al. (2023) [47] and Macias de la Rosa et al. (2023) [56] reported on the antiproliferative activity of organic extracts from the biomasses of *C. rotalis* and *H. akashiwo* (obtained with solvents of different polarities) against four human tumour lines. On the other hand, *A. carterae* is a well-known producer of bioactive substances, such as amphidinols, which exhibit potent antifungal, ichthyotoxic, haemolytic, cytotoxic, and antiprotozoal activities [65].

**Figure 6:**
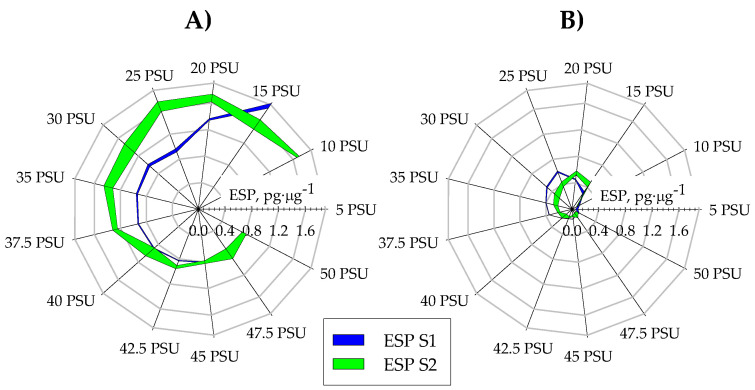
Haemolytic activity measured as equivalent saponin potential (ESP) expressed in saponin pg per μg of methanolic biomass extract in response to salinity variations in both Stage 1 (S1) and Stage 2 (S2) for the strains (**A**) *A. carterae* and (**B**) *C. rotalis*.

Measuring the haemolytic capacity of extracts obtained from microalgae has proven to be a good indicator of the presence of bioactive secondary metabolites [66], for which there are no primary quantification standards. Haemolytic activity measurements of the methanolic extracts from the three microalgae were carried out under the different conditions tested, the results of which are shown in Figure 6. The microalgae *H. akashiwo* showed no haemolytic activity in any of the trials performed, although it is known to produce bioactive compounds [4], even producing anaesthetic activity in fish [67]. Therefore, according to the bibliography and given that *H. akashiwo* showed no haemolytic activity, it seems appropriate to use this parameter as an indicator for assessing the presence of bioactives generated by this species. On the other hand, the maximum haemolytic activity values reached by *C. rotalis* were 0.55 ± 0.03 pg/μg at 20 PSU in S2 and 0.6 ± 0.01 pg/μg at 25 PSU in S1. Both haemolytic activity values are three times higher than those reported for a culture of *C. rotalis* grown in an 80 L tubular photobioreactor (T-PBR) [68]. This difference in the haemolytic activity values is probably due to the difference in salinity of the culture medium used in the T-PBR since it had a salinity of 30 PSU. When increasing the salt concentration in the medium from 40 PSU, no statistically significant differences (*p* < 0.05) were found in the ESP values of *C. rotalis*, fixing it at ESP values of 0.15 pg/μg and 0.1 pg/μg for S1 and S2, respectively. The maximum haemolytic activity values achieved by the *A. carterae* biomass (ESP = 1.68 pg/μg at 10 PSU in S1 and ESP = 1.88 pg/μg at 15 PSU in S2) are 5 times the haemolytic capacity shown by *C. rotalis* in our studies. As shown in Figure 4, at low salinities, the ESP values in *A. carterae* were the highest achieved, irrespective of the cultivation strategy followed. Regardless of the culture strategy followed, a decrease in ESP was observed as salinity increased, reaching an average value of 0.85 ± 0.05 (*p* < 0.05) in S1 for salinities above 40 PSU and 0.8 ± 0.05 (*p* < 0.05) in S2 for salinities greater than 45 PSU. These data indicate a strong dependence on environmental conditions, in this case, salinity, for the production of haemolytic compounds. Salinity-modulated production of bioactive compounds is a phenomenon observed in various microalgae. For example, several studies have evaluated the relationship between salinity and saxitoxin production in the dinoflagellate *Alexandrium*. Some of these studies identified higher saxitoxin productivity under salinity conditions that favour optimal growth [38], while others found that higher toxin production occurred at lower salinities [69]. In *Fibrocapsa japonica* [30], an increase in haemolytic activity was observed at salinities lower than those considered optimal for growth.

## 3. Conclusions

In this study, it was possible to successfully evaluate the culture of three microalgae species (*C. rotalis*, *A. carterae*, and *H. akashiwo*) using salinity as a factor to induce the production of high-value compounds, such as carotenoids, fatty acids, and bioactive compounds with haemolytic activity; this demonstrated that they are able to withstand various salinity levels from hyposaline conditions at 5 PSU to hypersaline conditions at 50 PSU. Biomass productivity was modelled with salinity in all three strains.

The salinity values that yielded the best *Pb* results in S1 were 30.58, 32.7, and 21.31 PSU (as determined by the proposed model) for *C. rotalis*, *A. carterae,* and *H. akashiwo*, respectively. *C. rotalis* reached its highest *P_CARO_* in S2 at a salinity of 20 PSU, while the best *P_FA_* values were similar across both strategies, recorded at 30 PSU (S1) and 20 PSU (S2). The maximum ESP value for *C. rotalis* was also achieved in S2 at 20 PSU. On the other hand, *A. carterae* achieved the highest *P_CARO_*, *P_FA_*, and ESP in S1 at salinities of 30 PSU, 25 PSU, and 15 PSU, respectively. *H. akashiwo* reached its best *P_CARO_* and *P_FA_* values in S2 at 35 PSU and 25 PSU, respectively, and showed no haemolytic activity in either S1 or S2.

In conclusion, a two-stage cultivation with salinity shifts led to increased concentrations of carotenoids, EPA, DHA, and haemolytic activity in *C. rotalis*, particularly in response to hyposaline shock. Similarly, applying a hyposaline shock in a two-stage cultivation of *A. carterae* enhanced the production of peridinin and haemolytic substances while also altering the fatty acid profile. In *H. akashiwo*, osmotic shock during two-stage cultivation resulted in higher levels of polyunsaturated fatty acids and carotenoids, such as fucoxanthin, at lower salinities. This method, two-stage cultivation with a saline shock, provides a promising framework for enhancing the production of several bioactive compounds such as carotenoids, fatty acids, and bioactive molecules with haemolytic activity from microalgae, offering valuable applications in the biotechnology industry.

## 4. Materials and Methods

### 4.1. Microalgae Species and Inoculums

The haptophyte *Chrysochromulina rotalis* BMCC18 (LT560338—GenBank accession number), the rhydophyte *Heterosigma akashiwo* BMCC75 (LC269919—GenBank accession number), and the dinoflagellate *Amphidinium carterae* Dn241EHAU (MG520273—GenBank accession number) provided by the Basque Microalgae Cultures Collection (BMCC) at the University of the Basque Country (UPV/EHU) were used. The microalgae were cultured in a thermostatized chamber at 18 ± 2 °C using a 75 cm^2^ T-flask (Nunc^TM^, EasYFlask^TM^, Thermo Scientific^TM^, Waltham, MA, USA) vertically arranged and artificially illuminated in 12 h:12 h light: dark cycles using 32 W fluorescent lamps (Master TL-D Eco 32W/840 SLV/25, Phillips, Eindhoven, The Netherlands) that provided a daily mean irradiance value of 150 μmol·m^−2^·s^−1^ on the culture surface. The culture medium was f/2 [70] with an N/P molar ratio of 5 [52] using filtered seawater (0.22 μm Whatman GF/F 47 mm filters; Maidstone, Kent, UK) with a salinity of 30 PSU adjusted by adding distilled water.

### 4.2. Optimal Salinity Conditions Established (S1)

*C. rotalis*, *H. akashiwo*, and *A. carterae* were cultured separately in discontinuous mode using culture media formulated with salinities between 5 PSU and 50 PSU; these cultures, which grew in one culture stage, will henceforth be denoted as S1. The experiments were carried out in a 75 cm^2^ T-flask (Nunc^TM^, EasYFlask^TM^, Thermo Scientific^TM^, Waltham, MA, USA) arranged vertically and filled to a height of 5 cm, meaning a working volume of 100 mL. The temperature and irradiance conditions used to grow the culture were those described in Section 4.1. Media with different salinities were prepared by diluting Mediterranean seawater with distilled water and/or adding sea salt (TorreSal, Unión salinera S.A, Alicante, Spain) until the desired saline concentration was achieved.

Cultured microalgae adapted to an f/2 environment with a salinity of 30 PSU were used as the inoculum. The cells used came from cultures that were in an exponential growth phase. The inoculum percentage used was around 10% of the work volume. After 22 days of cultivation, once the stationary growth phase was reached, the biomass was harvested via centrifugation (6000× *g*, 5 min; model SIGMA 4-15C,Osterode am Harz, Germany), and the pellets obtained were washed twice with a solution of 0.5 M of ammonium bicarbonate [71], then frozen for at least 48 h before being freeze-dried (Cryodos 50, Telstar, Terrassa, Spain).

### 4.3. Metabolic Response to Saline Shocks (S2)

Once the salinity value was obtained that maximized the biomass productivity (Section 4.2) of each of the three microalgae species, a new trial (S2) was undertaken in which each microalgae was cultured in batch mode with the optimal salinity obtained in S1 until the early stationary growth phase was reached; this was detected by a decrease in the specific growth rate and basal levels of macronutrients ((NO_3_)^−1^ and (PO_4_)^−3^) in the supernatant. At this point, the cells were harvested via centrifugation (200× *g* for *C. rotalis* and *H. akashiwo* and 300× *g* for *A. carterae* for 9–10 min) and resuspended in nutritionally limited culture media (10% of the nutrients in the f/2 medium with an N/P = 5 ratio) at different salinities (5 to 50 PSU). The cultures generated were kept under the same conditions (as specified in Section 4.2) for 48 h, after which they were harvested according to the protocol described in that section.

### 4.4. Kinetic Parameters

Biomass productivity (*Pb*), expressed in mg·L^−1^·day^−1^, was calculated using Equation (1). The yields of the different compounds (*Pp*) of interest studied (*P_CARO_*; carotenoids; *P_FA_*; fatty acids) were calculated using Equation (2), multiplying the biomass productivity by the percentage for the dry weight of each compound, *X*. The subscripts *i* and *f* refer to the start and end time of the culture.

(1) $$Pb=\frac{C_{bf}-C_{bi}}{t_{f}-t_{i}}$$

(2) $$P_{p}=Pb\cdot X$$

The final *Pb* values were correlated with the different salinities (*S*), tested using the following expression:

(3) $$Pb\left(S\right)=P_{max}+a\cdot (e^{\left(-{\left(\frac{S-S_{opt}}{b}\right)}^{2}\right)}-1)$$

where *a* (mg·L^−1^·day^−1^) and *b* (PSU) are adjustment parameters with no biological significance. *S_opt_* is the PSU salinity value that allows the maximum biomass productivity to be reached, *P_max_*, in mg·L^−1^·day^−1^.

The mean square error (*RMSE*) was determined to evaluate the accuracy of the fit between the actual data and those predicted by the proposed equation to study the growth or productivity as a function of the medium’s salinity.

(4) $$RMSE=\sqrt{\frac{\sum {\left(Pb_{p}-Pb_{e}\right)}^{2}}{n}}$$

where *Pb_p_* is the value predicted by the proposed equation and *Pb_P_* is the observed value, both expressed in mg·L^−1^·day^−1^, and *n* is the number of tests performed. Finally, to validate the proposed model, both the bias factor (*B_f_*) and the accuracy factor (*A_f_*) were evaluated; these were calculated using Equations (5) and (6), respectively [31].

(5) Bf=10∑logPbpPben

(6) Af=10∑logPbpPben

### 4.5. Analytical Measurements

Growth monitoring was performed via optical density measurement at 720 nm (OD_720_) (Biotek Synergy HT, Hampton, NH, USA). Prior to the tests described in this article, correlation lines were made between the dry weight of the different microalgae (*Cb*) and the OD_720_ values, a process previously described [72] for the three species of microalgae. The following lines were obtained: *Cb* (g·L^−1^) = 0.5905·OD_720_ (r^2^ = 0.915; *n* = 25) for *C. rotalis*; *Cb* (g·L^−1^) = 1.27·OD_720_ (r^2^= 0.97; *n* = 25) for *H. akashiwo*, and *Cb* (g·L^−1^) = 1.012·OD_720_ (r^2^ = 0.926; *n* = 56) for *A. carterae*, where *n* is the number of points used to make the lines. To assess the photosynthetic health of the microalgae, the maximum photosynthetic efficiency measurement of photosystem II (*Fv*/*Fm*) was routinely performed using a pulsed-light chlorophyll fluorometer (PAM-2500 Chlorophyll fluorometer, Heinz Walz GmbH; Effeltrich, Germany) according to the protocol described above [28]. The concentrations of macronutrients, nitrates, and phosphates were quantified in triplicate in the supernatant using spectrophotometric methods [52]. The carotenoid content and profile were determined using an HPLC photodiode array detector, as described by other authors [73], with 5 mg of freeze-dried biomass. The carotenoid pigments quantified were fucoxanthin, hex-fucoxanthin, 4-keto-hex-fucoxanthin, but-fucoxanthin, diadinoxanthin, diatoxanthin, diadinochrome, antheraxanthin, np. Chlc2-cp, peridinin, peridinol, peridinin ester, violaxanthin, zeaxanthin, and β-carotene. β-carotene and fucoxanthin standards from Sigma Chemical Co., were used (St. Louis, MO, USA), while the standards for violaxanthin, diadinoxanthin, diatoxanthin, diadinochrome, antheraxanthin, peridinin, and zeaxanthin came from DHI (Hørsholm, Denmark). With each of these standards, external calibration lines were established to quantify the concentrations. The de-epoxidation (*DES*) of diadinoxanthin (*Ddx*) to yield diatoxanthin (*Dtx*) [74], or that of violaxanthin (*Vx*) to yield zeaxanthin (*Zx*) [29], were calculated according to the following expressions:

(7) $$DES\left(\%\right)=\frac{Dtx}{Dtx+Ddx}\cdot 100$$

(8) $$DES\left(\%\right)=\frac{0.5\cdot Ax+Zx}{Vx+Ax+Zx}\cdot 100$$

where *Ddx*, *Dtx*, *Vx*, *Ax*, and *Zx* are the concentrations of diadinoxanthin, diatoxanthin, violaxanthin, antheraxanthin, and zeaxanthin, respectively, expressed in % d.w. The fatty acid profiles and contents were obtained using 10 mg of lyophilized biomass by direct transesterification and subsequent gas chromatography analysis (6890 N Series Gas Chromatograph, Agilent Technologies, Santa Clara, CA, USA), as described in [75]. Finally, the haemolytic activity of the biomass’s methanolic extracts [72] at EC50 values for saponin relative to the EC50 values for *C. rotalis* and *A. carterae* (called the equivalent saponin potential; ESP) was expressed in *ρ*g of saponin per μg of biomass methanolic extract that achieves 50% hemolysis in red blood cells.

### 4.6. Statistical Analysis

The experimental results are shown as the mean values of the two independent experiments and the replicates of the analyses and their standard deviation. Statistical data analyses were performed using Statgraphics Centurion XVII (version 17.2.04) statistical software (2014, Statpoint Technologies, Inc., Warrenton, VA, USA). Statgraphics was used for the analysis of significant differences through a single-variable simple analysis of variance (ANOVA) test, as well as for the calculation of sample homogeneity using the Levene test.

## Figures and Tables

**Table 1:** Initial (0) and final (F) concentrations of nitrates ([NO_3_]^−1^) and phosphates ([PO_4_]^−3^) in the culture medium for the different salinities for the three microalgae species used in S1.

Microalgae	Salinity, PSU	[NO_3_]_0_^−1^, μM	[NO_3_]_F_^−1^, μM	[PO_4_]_0_^−3^, μM	[PO_4_]_F_^−3^, μM
*C. rotalis*	5	893.62 ± 2.61	1.92 ± 0.20	176.43 ± 0.05	0.00 ± 0.00
	10	897.66 ± 1.87	0.88 ± 0.02	174.71 ± 0.23	0.61 ± 0.05
	20	882.41 ± 5.23	1.08 ± 0.05	180.12 ± 0.72	0.12 ± 0.01
	30	880.03 ± 0.12	1.18 ± 0.24	182.34 ± 0.05	0.00 ± 0.00
	40	893.60 ± 2.16	0.24 ± 0.03	180.00 ± 0.60	1.03 ± 0.05
	50	872.42 ± 0.83	0.88 ± 0.05	183.70 ± 1.45	0.47 ± 0.03
*A. carterae*	5	873.10 ± 3.25	0.46 ± 0.10	181.09 ± 0.05	0.55 ± 0.09
	10	887.66 ± 5.57	2.08 ± 0.02	179.22 ± 0.03	0.10 ± 0.05
	20	892.41 ± 3.26	0.08 ± 0.02	180.04 ± 1.23	0.00 ± 0.00
	30	883.69 ± 3.57	2.98 ± 0.54	181.11 ± 0.08	0.00 ± 0.00
	40	893.45 ± 7.44	0.00 ± 0.00	189.10 ± 0.00	0.00 ± 0.00
	50	880.66 ± 4.10	0.18 ± 0.02	186.65 ± 0.02	0.00 ± 0.03
*H. akashiwo*	5	883.09 ± 6.14	0.00 ± 0.00	188.66 ± 0.75	0.00 ± 0.00
	10	877.43 ± 1.00	0.03 ± 0.01	189.03 ± 3.03	0.06 ± 0.00
	20	889.07 ± 2.49	0.01 ± 0.00	182.25 ± 1.27	1.23 ± 0.01
	30	880.47 ± 0.66	0.18 ± 0.01	180.05 ± 0.25	0.72 ± 0.01
	40	878.23 ± 7.06	0.59 ± 0.03	184.70 ± 4.09	0.09 ± 0.01
	50	882.29 ± 4.03	0.00 ± 0.00	183.00 ± 1.75	1.98 ± 0.06

**Table 2:** Comparison of predicted and observed values in the growth of different microalgae as a function of the culture with medium salinity.

Microalgae	*RMSE*	*A_f_*	*B_f_*	*r* ^2^	(Ref.)
*C. rotalis*	0.434	1.027	1.002	0.953	This work
*A. carterae*	1.051	1.184	1.110	0.938	This work
*H. akashiwo*	0.397	1.044	1.003	0.938	This work
*A. pacificum*	1.059	1.000	0.999	0.999	[38]
*Desmodesmus* sp.	0.018	1.032	0.997	0.977	[39]
*A. platensis*	0.010	1.005	1.049	0.972	[40]

## Data Availability

The original contributions presented in the study are included in the article, further inquiries can be directed to the corresponding author.

## References

[1] Daneshvar E., Wicker R.J., Show P.-L., Bhatnagar A. (2022). Biologically-mediated carbon capture and utilization by microalgae towards sustainable CO_2_ biofixation and biomass valorization—A review. Chem. Eng. J..

[2] Allewaert C.C., Vanormelingen P., Daveloose I., Verstraete T., Vyverman W. (2017). Intraspecific trait variation affecting astaxanthin productivity in two *Haematococcus* (Chlorophyceae) species. Algal Res..

[3] Shetty P., Gitau M.M., Maróti G. (2019). Salinity stress responses and adaptation mechanisms in eukaryotic green microalgae. Cells.

[4] Tang D.Y.Y., Khoo K.S., Chew K.W., Tao Y., Ho S.-H., Show P.L. (2020). Potential utilization of bioproducts from microalgae for the quality enhancement of natural products. Bioresour. Technol..

[5] Assunção J., Guedes A.C., Malcata F.X. (2017). Biotechnological and Pharmacological Applications of Biotoxins and Other Bioactive Molecules from Dinoflagellates. Mar. Drugs.

[6] Chisti Y. (2007). Biodiesel from microalgae. Biotechnol. Adv..

[7] Lafarga T., Acién G. (2022). Microalgae for the Food Industry: From Biomass Production to the Development of Functional Foods. Foods.

[8] Abdelfattah A., Ali S.S., Ramadan H., El-Aswar E.I., Eltawab R., Ho S.H., Elsamahy T., Li S., El-Sheekh M.M., Schagerl M. (2023). Microalgae-based wastewater treatment: Mechanisms, challenges, recent advances, and future prospects. Environ. Sci. Ecotechnol..

[9] Maurya R., Zhu X., Valverde-Pérez B., Ravi Kiran B., General T., Sharma S., Kumar Sharma A., Thomsen M., Venkata Mohan S., Angelidaki I. (2021). Advances in microalgal research for valorization of industrial wastewater. Bioresour. Technol..

[10] Farrag M.M.S., Abdelmgeed A.M., Moustafa M.A., Osman A.G.M. (2024). Improving the water quality of fish aquaculture effluents after treatment by microalgae. Desalin. Water Treat..

[11] Yaacob N.S., Abdullah H., Ahmad M.F., Maniyam M.N., Sjahrir F. (2022). Microalgae Biotechnology: Emerging Biomedical Applications.

[12] Esakkimuthu S., Siddiqui S.A., Cherif M., Saadaoui I. (2024). Exploring strategies to enhance microalgae nutritional quality for functional poultry-sourced food products. Bioresour. Technol. Rep..

[13] Ishika T., Bahri P.A., Laird D.W., Moheimani N.R. (2018). The effect of gradual increase in salinity on the biomass productivity and biochemical composition of several marine, halotolerant, and halophilic microalgae. J. Appl. Phycol..

[14] Kirst G.O. (1990). Salinity tolerance of eukaryotic marine algae. Annu. Rev. Plant Physiol. Plant Mol. Biol..

[15] Ren Y., Sun H., Deng J., Huang J., Chen F. (2021). Carotenoid Production from Microalgae: Biosynthesis, Salinity Responses and Novel Biotechnologies. Mar Drugs..

[16] Zittelli G.C., Lauceri R., Faraloni C., Benavides A.M.S., Torzillo G. (2023). Valuable pigments from microalgae: Phycobiliproteins, primary carotenoids, and fucoxanthin. Photochem. Photobiol. Sci..

[17] Ben-Amotz A., Sussman I., Avron M., Mislin H., Bachofen R. (1982). Glycerol production by Dunaliella. New Trends in Research and Utilization of Solar Energy through Biological Systems.

[18] Hellebust J.A. (1976). Effect of salinity on photosynthesis and mannitol synthesis in the green flagellate Platymonas suecica. Botany.

[19] Wang H.L., Postier B.L., Burnap R.L. (2002). Polymerase chain reaction-based mutageneses identify key transporters belonging to multigene families involved in Na+ and pH homeostasis of *Synechocystis* sp. PCC 6803. Mol. Microbiol..

[20] Singh R.P., Yadav P., Kumar A., Hashem A., Avila-Quezada G.D., Abd_Allah E.F., Gupta R.K. (2023). Salinity-Induced Physiochemical Alterations to Enhance Lipid Content in Oleaginous Microalgae *Scenedesmus* sp. BHU1 via Two-Stage Cultivation for Biodiesel Feedstock. Microorganisms.

[21] Flores-Leñero A., Vargas-Torres V., Paredes-Mella J., Norambuena L., Fuenzalida G., Lee-Chang K., Mardones J.I. (2022). *Heterosigma akashiwo* in Patagonian Fjords: Genetics, Growth, Pigment Signature and Role of PUFA and ROS in Ichthyotoxicity. Toxins.

[22] Liyanaarachchi V.C., Premaratne M., Ariyadasa T.U., Nimarshana P.H.V., Malik A. (2021). Two-stage cultivation of microalgae for production of high-value compounds and biofuels: A review. Algal Res..

[23] Tafreshi A.H., Shariati M. (2006). Pilot culture of three strains of *Dunaliella salina* for β-carotene production in open ponds in the central region of Iran. World J. Microbiol. Biotechnol..

[24] Xia L., Ge H., Zhou X., Zhang D., Hu C. (2013). Photoautotrophic outdoor two-stage cultivation for oleaginous microalgae Scenedesmus obtusus XJ-15. Bioresour. Technol..

[25] Chen H.-H., Xue L.-L., Liang M.-H., Jiang J.-G. (2019). Sodium azide intervention, salinity stress and two-step cultivation of Dunaliella tertiolecta for lipid accumulation. Enzyme Microb. Technol..

[26] Rearte T.A., Figueroa F.L., Gómez-Serrano C., Vélez C.G., Marsili S., Iorio A.d.F., González-López C.V., Cerón-García M.C., Abdala R., Acién F.G. (2020). Optimization of the production of lipids and carotenoids in the microalga Golenkinia aff. Brevispicula. Algal Res..

[27] Eggert A., Raimund S., Michalik D., West J., Karsten U. (2007). Ecophysiological performance of the primitive red alga *Dixoniella grisea* (Rhodellophyceae) to irradiance, temperature and salinity stress: Growth responses and the osmotic role of mannitol. Phycol..

[28] López-Rosales L., Gallardo-Rodríguez J.J., Sánchez-Mirón A., Cerón-García M.D.C., Belarbi E.H., García-Camacho F., Molina-Grima E. (2014). Simultaneous effect of temperature and irradiance on growth and okadaic acid production from the marine dinoflagellate Prorocentrum belizeanum. Toxins.

[29] Torzillo G., Faraloni C., Silva A.M., Kopecký J., Pilný J., Masojídek J. (2012). Photoacclimation of *Phaeodactylum tricornutum* (Bacillariophyceae) cultures grown outdoors in photobioreactors and open ponds. Eur. J. Phycol..

[30] Darvehei P., Bahri P.A., Moheimani N.R. (2018). Model development for the growth of microalgae: A review. Renew. Sustain. Energy Rev..

[31] De Boer M., Tyl M.R., van Rijssel M. (2004). Effects of salinity and nutrient conditions on growth and haemolytic activity of *Fibrocapsa japonica* (Raphidophyceae). Aquat. Microb. Ecol..

[32] Xing S., Zhang X., Guan H., Li H., Liu W. (2022). Predictive model for growth of Leuconostoc mesenteroides in Chinese cabbage juices with different salinities. LWT.

[33] Mehmet K., Mehmet A. (2005). Studies on Growth of Marine Microalgae in Batch Cultures: II. *Isochrysis galbana* (Haptophyta). Asian J. Plant Sci..

[34] Morton S.L., Norris D.R., Bomber J.W. (1992). Effect of temperature, salinity and light intensity on the growth and seasonality of toxic dinoflagellates associated with ciguatera. J. Exp. Mar. Bio. Ecol..

[35] Hotos G.N., Avramidou D. (2021). The Effect of Various Salinities and Light Intensities on the Growth Performance of Five Locally Isolated Microalgae [*Amphidinium carterae*, *Nephroselmis* sp., *Tetraselmis* sp. (var. red pappas), *Asteromonas gracilis* and *Dunaliella* sp.] in Laboratory Batch Cultures. J. Mar. Sci. Eng..

[36] Haque S.M., Onoue Y. (2002). Effects of salinity on growth and toxin production of a noxious phytoflagellate, Heterosigma akashiwo (Raphidophyceae). Bot. Mar..

[37] Martínez R., Orive E., Laza-Martínez A., Seoane S. (2010). Growth response of six strains of Heterosigma akashiwo to varying temperature, salinity and irradiance conditions. J. Plankton Res..

[38] Bui Q.T.N., Kim H., Park H., Ki J.-S. (2021). Salinity Affects Saxitoxins (STXs) Toxicity in the Dinoflagellate *Alexandrium pacificum*, with Low Transcription of SXT-Biosynthesis Genes *sxtA4* and *sxtG*. Toxins.

[39] Mehariya S., Plöhn M., Leon-Vaz A., Patel A., Funk C. (2022). Improving the content of high value compounds in Nordic Desmodesmus microalgal strains. Bioresour. Technol..

[40] Yu C., Hu Y., Zhang Y., Luo W., Zhang J., Xu P., Qian J., Li J., Yu J., Liu J. (2024). Concurrent enhancement of biomass production and phycocyanin content in salt-stressed *Arthrospira platensis*: A glycine betaine- supplementation approach. Chemosphere.

[41] Moriasi D.N., Arnold J.G., Liew M., Bingner R.L., Harmel R.D., Veith T.L. (2007). Model Evaluation Guidelines for Systematic Quantification of Accuracy in Watershed Simulations. Trans. ASABE.

[42] Ross T. (1996). Indices for performance evaluation of predictive models in food microbiology. J. Appl. Bacteriol..

[43] Paliwal C., Mitra M., Bhayani K., Bharadwaj S.V.V., Ghosh T., Dubey S., Mishra S. (2017). Abiotic stresses as tools for metabolites in microalgae. Bioresour. Technol..

[44] Seoane S., Zapata M., Orive E. (2009). Growth rates and pigment patterns of haptophytes isolated from estuarine waters. J. Sea Res..

[45] Krinsky N.I., Johnson E.J. (2005). Carotenoid actions and their relation to health and disease. Mol. Asp. Med..

[46] Muthuirulappan S., Francis S.P. (2013). Anti-cancer mechanism and possibility of nano-suspension formulations for a marine algae product fucoxanthin. Asian Pac. J. Cancer Prev..

[47] González-Cardoso M., Cerón-García M., Navarro-López E., Molina-Miras A., Sánchez-Mirón A., Contreras-Gómez A., García-Camacho F. (2023). Alternatives to Classic Solvents for the Isolation of Bioactive Compounds from Chrysochromulina rotalis. Bioresour. Technol..

[48] Smaoui S., Barkallah M., Ben Hlima H., Fendri I., Khaneghah A.M., Michaud P., Abdelkafi S. (2021). Microalgae Xanthophylls: From Biosynthesis Pathway and Production Techniques to Encapsulation Development. Foods.

[49] Zhao Y., Wang H.-P., Han B., Yu X. (2019). Coupling of abiotic stresses and phytohormones for the production of lipids and high-value by-products by microalgae: A review. Bioresour. Technol..

[50] Zapata M., Fraga S., Rodríguez F., Garrido J. (2012). Pigment-based chloroplast types in dinoflagellates. Mar. Ecol. Prog. Ser..

[51] Johansen J.E., Svec W.A., Liaaen-Jensen S., Haxo F.T. (1974). Carotenoids of the dinophyceae. Phytochemistry.

[52] Molina-Miras A., López-Rosales L., Sánchez-Mirón A., Cerón-García M., Seoane-Parra S., García-Camacho F., Molina-Grima E. (2018). Long-term culture of the marine dinoflagellate microalga Amphidinium carterae in an indoor LED-lighted raceway photobioreactor: Production of carotenoids and fatty acids. Bioresour. Technol..

[53] Kichouh-Aiadi S., Gallardo-Rodríguez J.J., Cerón-García M.C., López-Rosales L., García-Camacho F., Sánchez-Mirón A. (2024). Exploring the potential of epigenetic chemicals to increase metabolite production in the dinoflagellate microalga Amphidinium carterae. J.Appl. Phycol..

[54] Ruivo M., Amorim A., Cartaxana P. (2011). Effects of growth phase and irradiance on phytoplankton pigment ratios: Implications for chemotaxonomy in coastal waters. J. Plankton Res..

[55] Latasa M., Berdalet E. (1994). Effect of nitrogen or phosphorus starvation on pigment composition of cultured *Heterocapsa* sp. J. Plankton Res..

[56] Macías-de la Rosa A., González-Cardoso M.Á., Cerón-García M.d.C., López-Rosales L., Gallardo-Rodríguez J.J., Seoane S., Sánchez-Mirón A., García-Camacho F. (2023). Bioactives Overproduction through Operational Strategies in the Ichthyotoxic Microalga Heterosigma akashiwo Culture. Toxins.

[57] Haris N., Manan H., Jusoh M., Khatoon H., Katayama T., Kasan N.A. (2022). Effect of different salinity on the growth performance and proximate composition of isolated indigenous microalgae species. Aquac. Rep..

[58] Ra C.H., Kang C.-H., Kim N.K., Lee C.-G., Kim S.-K. (2015). Cultivation of four microalgae for biomass and oil production using a two-stage culture strategy with salt stress. Renew. Energy.

[59] Bigelow N., Barker J., Ryken S., Patterson J., Hardin W., Barlow S., Deodato C., Cattolico R.A. (2013). *Chrysochromulina* sp.: A proposed lipid standard for the algal biofuel industry and its application to diverse taxa for screening lipid content. Algal Res. -Biomass Biofuels Bioprod..

[60] Khoeyi Z.A., Seyfabadi J., Ramezanpour Z. (2011). Effect of light intensity and photoperiod on biomass and fatty acid composition of the microalgae, Chlorella vulgaris. Aquac. Int..

[61] Reitan K.I., Rainuzzo J.R., Olsen Y. (1994). Effect of nutrient limitation on fatty acid and lipid content of marine microalgae. J. Phycol..

[62] Fal S., Aasfar A., Rabie R., Smouni A., Arroussi H.E. (2022). Salt induced oxidative stress alters physiological, biochemical and metabolomic responses of green microalga *Chlamydomonas reinhardtii*. Heliyon.

[63] Mansour M.P., Frampton D.M.F., Nichols P.D., Volkman J.K., Blackburn S.I. (2005). Lipid and fatty acid yield of nine stationary-phase microalgae: Applications and unusual C24–C28 polyunsaturated fatty acids. J. Appl. Phycol..

[64] Molina-Miras A., López-Rosales L., Sánchez-Mirón A., López-Rodríguez M., Cerón-García M., García-Camacho F., Molina-Grima E. (2020). Influence of culture medium recycling on the growth of a marine dinoflagellate microalga and bioactives production in a raceway photobioreactor. Algal Res..

[65] Kobayashi J., Kubota T. (2010). Bioactive metabolites from marine dinoflagellates. Comprehensive Natural Products II.

[66] Abreu A.C., Molina-Miras A., Aguilera-Sáez L.M., López-Rosales L., Cerón-García M.d.C., Sánchez-Mirón A., Olmo-García L., Carrasco-Pancorbo A., García-Camacho F., Molina-Grima E. (2019). Production of Amphidinols and Other Bioproducts of Interest by the Marine Microalga *Amphidinium carterae* Unraveled by Nuclear Magnetic Resonance Metabolomics Approach Coupled to Multivariate Data Analysis. J. Agric. Food Chem..

[67] Gallardo-Rodríguez J., Astuya-Villalón A., Avello V., Llanos-Rivera A., Krock B., Agurto-Muñoz C., Sánchez-Mirón A., García-Camacho F. (2020). Production of extracts with anaesthetic activity from the culture of Heterosigma akashiwo in pilot-scale photobioreactors. Algal Res..

[68] La Rosa A.M.-D., López-Rosales L., Cerón-García M., Molina-Miras A., Soriano-Jerez Y., Sánchez-Mirón A., Seoane S., García-Camacho F. (2023). Assessment of the marine microalga Chrysochromulina rotalis as bioactive feedstock cultured in an easy-to-deploy light-emitting-diode-based tubular photobioreactor. Bioresour. Technol..

[69] Lim P.T., Ogata T. (2005). Salinity effect on growth and toxin production of four tropical *Alexandrium* species (Dinophyceae). Toxicon.

[70] Guillard R.R., Ryther J.H. (1962). Studies of marine planktonic diatoms. I. cyclotella nana Hustedt, and Detonula confervacea (cleve). Gran. Can. J. Microbiol..

[71] Zhu C.J., Lee Y.K. (1997). Determination of biomass dry weight of marine microalgae. J. Appl. Phycol..

[72] Molina-Miras A., Morales-Amador A., de Vera C., López-Rosales L., Sánchez-Mirón A., Souto M., Fernández J., Norte M., García-Camacho F., Molina-Grima E. (2018). A pilot-scale bioprocess to produce amphidinols from the marine microalga Amphidinium carterae: Isolation of a novel analogue. Algal Res..

[73] Cerón-García M.C., González-López C.V., Camacho-Rodríguez J., López-Rosales L., García-Camacho F., Molina-Grima E. (2018). Maximizing carotenoid extraction from microalgae used as food additives and determined by liquid chromatography (HPLC). Food Chem..

[74] Hemker F., Zielasek F., Jahns P. (2024). Combined high light and salt stress enhances accumulation of PsbS and zeaxanthin in Chlamydomonas reinhardtii. Physiol. Plant..

[75] Rodríguez-Ruiz J., Belarbi E.-H., Sánchez J.L.G., Alonso D.L. (1998). Rapid simultaneous lipid extraction and transesterification for fatty acid analyses. Biotechnol. Tech..

